# Antiviral Sulfoquinovosyldiacylglycerols (SQDGs) from the Brazilian Brown Seaweed *Sargassum vulgare*

**DOI:** 10.3390/md11114628

**Published:** 2013-11-21

**Authors:** Erwan Plouguerné, Lauro M. de Souza, Guilherme L. Sassaki, Jéssica Figueiredo Cavalcanti, Maria Teresa Villela Romanos, Bernardo A. P. da Gama, Renato Crespo Pereira, Eliana Barreto-Bergter

**Affiliations:** 1 Department of Marine Biology, Biology Institute, Fluminense Federal University, Niterói 24210-130, RJ, Brazil; E-Mails: bapgama@pq.cnpq.br (B.A.P.G.); rcrespo@id.uff.br (R.C.P.); 2 Department of Biochemistry and Molecular Biology, Paraná Federal University, Curitiba 81531-990, PR, Brazil; E-Mails: laurosouza@hotmail.com (L.M.S.); sassaki@ufpr.br (G.L.S.); 3 Department of Virology, Microbiology Institute, Federal University of Rio de Janeiro, Rio de Janeiro 21941-902, RJ, Brazil; E-Mails: jessica.f.cavalcanti@hotmail.com (J.F.C.); teresa.romanos@micro.ufrj.br (M.T.V.R.); 4 Department of General Microbiology, Microbiology Institute, Federal University of Rio de Janeiro, Rio de Janeiro 21941-902, RJ, Brazil; E-Mail: eliana.bergter@micro.ufrj.br

**Keywords:** *Sargassum vulgare*, glycolipids, sulfoquinovosyldiacylglycerol, anti-HSV activity, palmitic acid

## Abstract

Total lipids from the Brazilian brown seaweed *Sargassum vulgare* were extracted with chloroform/methanol 2:1 and 1:2 (v/v) at room temperature. After performing Folch partition of the crude lipid extract, the lipids recovered from the Folch lower layer were fractionated on a silica gel column eluted with chloroform, acetone and methanol. The fraction eluted with methanol, presented a strong orcinol-positive band characteristic of the presence of sulfatides when examined by TLC. This fraction was then purified by two successive silica gel column chromatography giving rise to fractions F4I86 and F4II90 that exhibited strong activity against herpes simplex virus type 1 and 2. The chemical structures present in both fractions were elucidated by ESI-MS and ^1^H/^13^C NMR analysis HSQC fingerprints based on their tandem–MS behavior as sulfoquinovosildiacylglycerols (SQDGs). The main SQDG present in both fractions and responsible for the anti-herpes activity observed was identified as 1,2-di-*O*-palmitoyl-3-*O*-(6-sulfo-α-d-quinovopyranosyl)-glycerol.

## 1. Introduction

Herpes simplex virus 1 (HSV-1) and herpes simplex virus 2 (HSV-2) are the most widely studied human herpes viruses [1] with an estimated 60%–95% of the adult population infected by at least one of them [1,2]. HSV-1 is generally related to oral–facial infections and encephalitis, whereas HSV-2 is responsible for genital infections, and can be transferred from infected mothers to neonates [1]. Moreover, HSV infections are recognized as a risk factor for human immunodeficiency virus (HIV) infection [3]. Efficient anti-herpes drugs already exist, but their extensive use can generate side effects and may also lead to the rise of drug-resistant virus strains [4,5]. Consequently, new types of anti-herpes compounds are urgently needed.

Marine organisms are a huge source of natural products with biological activities. Products of primary metabolism like amino acids, carbohydrates and proteins, are vital for maintaining life processes, while others such as alkaloids, phenolics, steroids, terpenoids, are secondary metabolites that have ecological, toxicological and pharmacological significance [6,7], encompassing bioactivities such as antiparasitic, antitumor, antimicrobial and antifoulant effects [8].

Recently, a great deal of interest has been expressed regarding compounds from seaweeds as potential antiviral agents [9]. Polysaccharides (sulfated polysaccharides in particular), poliketides, terpenoids or peptides with anti-HSV activities have been isolated from these marine organisms [10,11,12,13]. Glycolipids represent a less studied class of antiviral secondary metabolites [14]. Seaweeds synthesize three major types of glycolipids: monogalactosyldiacylglycerides (MGDG), digalactosyldiacylglycerides (DGDG), and sulfoquinovosyldiacylglycerides (SQDG) [15]. SQDG has an important biological function in photosynthetic plant tissues [16], exhibits high biological activity [17], affects HIV [18] and neoplastic and inflammatory processes [17,19]. In a recent study, de Souza and coworkers [14] isolated SQDG from the red seaweed *Osmundaria obtusiloba* that exhibited potent anti-HSV-1 and HSV-2 activities. A SQDG with anti-HSV-1 activity was isolated from the microalga *Spirula platensis* [20]. Wang and coworkers [21] highlighted the anti-HSV-2 activity of a SQDG isolated from the green seaweed *Caulerpa racemosa*. As SQDG is the main glycolipid found in brown seaweeds of the order Fucales [22], we have chosen the brown seaweed *Sargassum vulgare* as a model in order to isolate and test its glycolipids as potencial anti-HSV-1 and HSV-2 agents.

## 2. Results and Discussion

### 2.1. Lipid Fractionation

**Figure 1:**
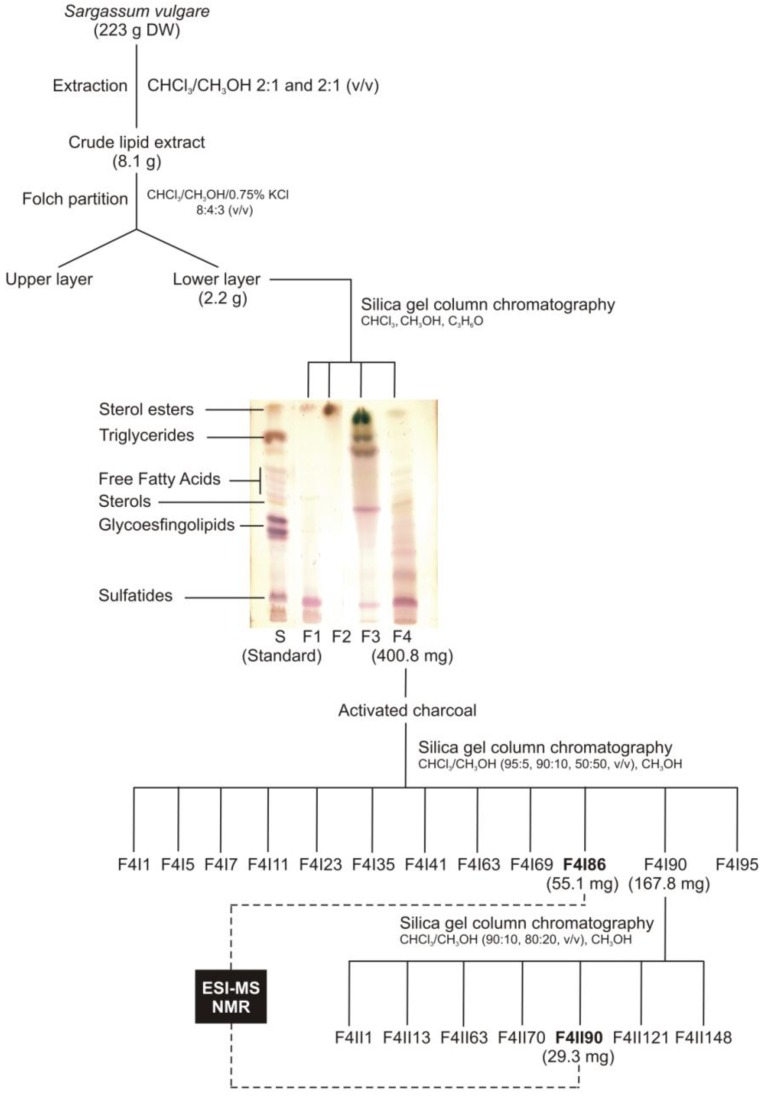
Purification protocol of sulfoquinovosyldiacylglycerols from *Sargassum vulgare*.

Total lipids from the brown seaweed *Sargassum vulgare* were successively extracted with chloroform/methanol 2:1 and 1:2 (v/v) at room temperature according to previous studies [14,23]. After filtration, the extracts were combined, concentrated in vacuo and the crude lipid extract was partitioned according to Folch and coworkers [24]. The lower layer was evaporated and fractionated on silica gel column chromatography using chloroform, acetone, and methanol as solvents (Figure 1). Fractions were analyzed by TLC, developed with CHCl_3_:CH_3_OH:2M NH_4_OH (40:10:1 v/v/v) and the spots visualized with iodine and by spraying with orcinol/H_2_SO_4_ [23]. The resulting fractions were combined in four fractions, F1, F2, F3 and F4 according to their TLC profiles. Thin-layer chromatography of F4 revealed an orcinol-positive band with chromatographic mobility corresponding to a sulfatide. This fraction was then chosen to carry out the purification protocol.

The F4 fraction was first treated with activated charcoal in order to remove the pigments, and was then purified on a silica gel column, which was sequentially eluted with chloroform/methanol with increasing concentrations of methanol (95:5, 90:10, 80:20, 50:50, v/v) and finally with 100% methanol, providing ninety-five sub-fractions. These fractions were pooled according to their TLC profiles, resulting in twelve final fractions: F4I1, F4I5, F4I7, F4I11, F4I23, F4I35, F4I41, F4I63, F4I69, F4I86, F4I90 and F4I95.

Fraction F4I90 was further purified on a silica gel column, which was sequentially eluted with chloroform/methanol with increasing concentrations of methanol (90:10, 80:20 v/v) and finally with 100% methanol, providing hundred fifty-one sub-fractions. These fractions were pooled according to their TLC profiles, resulting in seven final fractions: F4II1, F4II13, F4II63, F4II70, F4II90, F4II121, and F4II148.

Fractions F4I86 and F4II90, which TLC profiles indicate the presence of SQDGs, were then analyzed using ESI-MS and NMR, and their antiviral activity was tested against HSV-1 and HSV-2.

### 2.2. Mass Spectrometry of Sulfolipids

The spectrum obtained in negative MS^1^ from fraction F4I86 exhibited six deprotonated ions with *m/z* 766, 794, 808, 820, 836 and 892 [M − H]^−^ compatible with sulfoquinovosyldiacylglycerol structures.

**Figure 2:**
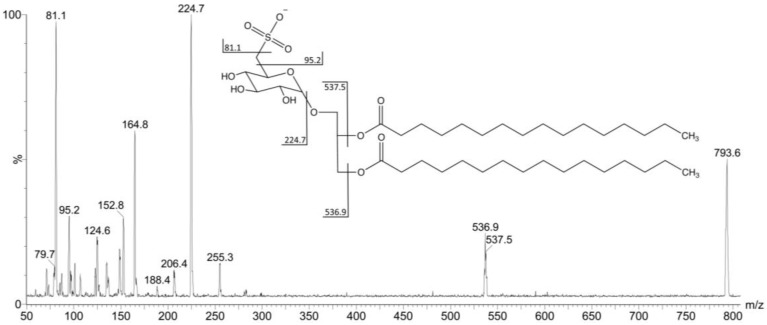
Spectrum from MS^1^ obtained in negative ionization mode from Fraction F4I86. The fragmentation pathway of the ion at *m/z* = 794 is compatible with the structure of 1,2-di-*O*-palmitoyl-3-*O*-(6-sulfoquinovopyranosyl)-glycerol.

In order to confirm the structures, the ions at *m/z* 766, 794, 808, 820, 836 and 892 were fragmented by the second stage tandem-MS. Each ion gave fragments at *m/z* 225, 165, 153, 95 and 81 characteristic of the 6-deoxy-6-sulfono-hexosyl residue of the SQDG (Figure 2).

The ion at *m/z* 793.9 was the most abundant and gave fragments at *m/z* 537.5 (M − C16:0 from the *sn*-2 position), 536.9 (M–C16:0 from the *sn*-1 position), 224.7, 164.8, 152.8, 95.2 and 81.1, as indicated in the fragmentation pathway, consistent with a SQDG structure, esterified by two palmitic acids (C16:0) (Figure 2). The structure was confirmed comparing our data to the fragmentation pathway already described by Zianni and coworkers [25] for a similar SQDG isolated in a lipid extract from spinach leaves.

**Figure 3:**
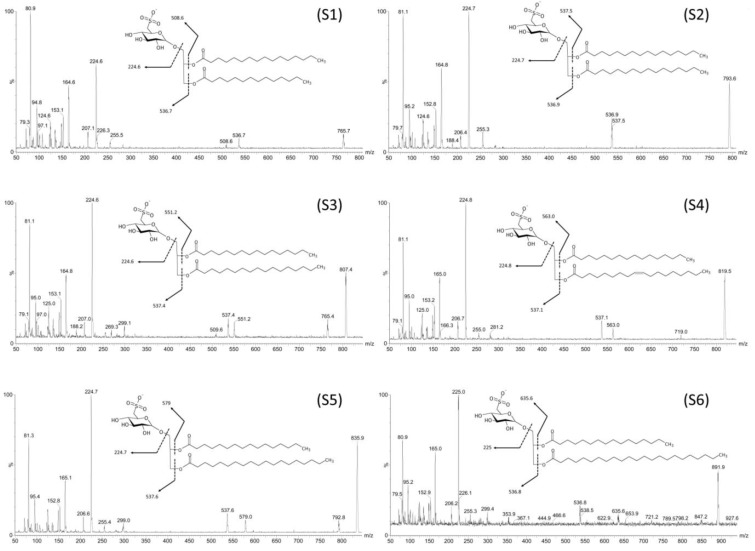
Characteristic tandem-MS (MS^1^) profiles obtained from SQDGs isolated from fractions F4I86 and F4II90. The fragmentation indicated differences in the fatty acid chain lengths and the putative structures were depicted for each SQDG species. (**S1**) 1-*O*-myristoyl-2-*O*-palmitoyl-3-*O*-(6-sulfoquinovopyranosyl)-glycerol, (**S2**) 1,2-di-*O*-palmitoyl-3-*O*-(6-sulfoquinovopyranosyl)-glycerol, (**S3**) 1-*O*-margaroyl-2-*O*-palmitoyl-3-*O*-(6-sulfoquinovopyranosyl)-glycerol, (**S4**) 1-*O*-oleoyl-2-*O*-palmitoyl-3-*O*-(6-sulfoquinovopyranosyl)-glycerol, (**S5**) 1-*O*-nonadecanoyl-2-*O*-palmitoyl-3-*O*-(6-sulfoquinovopyranosyl)-glycerol, (**S6**) 1-*O*-tricosanoyl-2-*O*-margaroyl-3-*O*-(6-sulfoquinovopyranosyl)-glycerol.

The fragmentation pathway of the six deprotonated ions with *m/z* at 765.7, 793.6, 807.4, 819.5, 835.9 and 891.9 [M − H]^−^, is compatible with sulfoquinovosyldiacylglycerol structures represented in Table 1 and Figure 3.

**Table 1:** Identification of sulfoquinovosyldiacylglycerides (SQDGs) present in fractions F4I86 and F4II90.

Fraction	Compound	R1/R2	[M − H]^−^ *m/z*	[M − R1]^−^ *m/z*	[M − R2]^−^ *m/z*
F4I86, F4II90	S1	C_14:0_/C_16:0_	765.7	536.7	508.6
F4I86, F4II90	S2	C_16:0_/C_16:0_	793.6	537	537
F4I86, F4II90	S3	C_17:0_/C_16:0_	807.4	537.4	551.2
F4I86	S4	C_18:1_/C_16:0_	819.5	537.1	563
F4I86, F4II90	S5	C_19:0_/C_16:0_	835.9	537.6	579
F4I86	S6	C_23:0_/C_17:0_	891.9	536.8	635.6

The spectrum obtained from fraction F4II90 presents the same ions that fraction F4I86, with the exception of the ions at *m/z* = 819.5 and *m/z* = 891.9 that correspond to SQDG structures esterified by palmitic and oleic acids, and by palmitic and tricosanoic acids respectively.

### 2.3. NMR Spectroscopy of Sulfolipids

**Figure 4:**
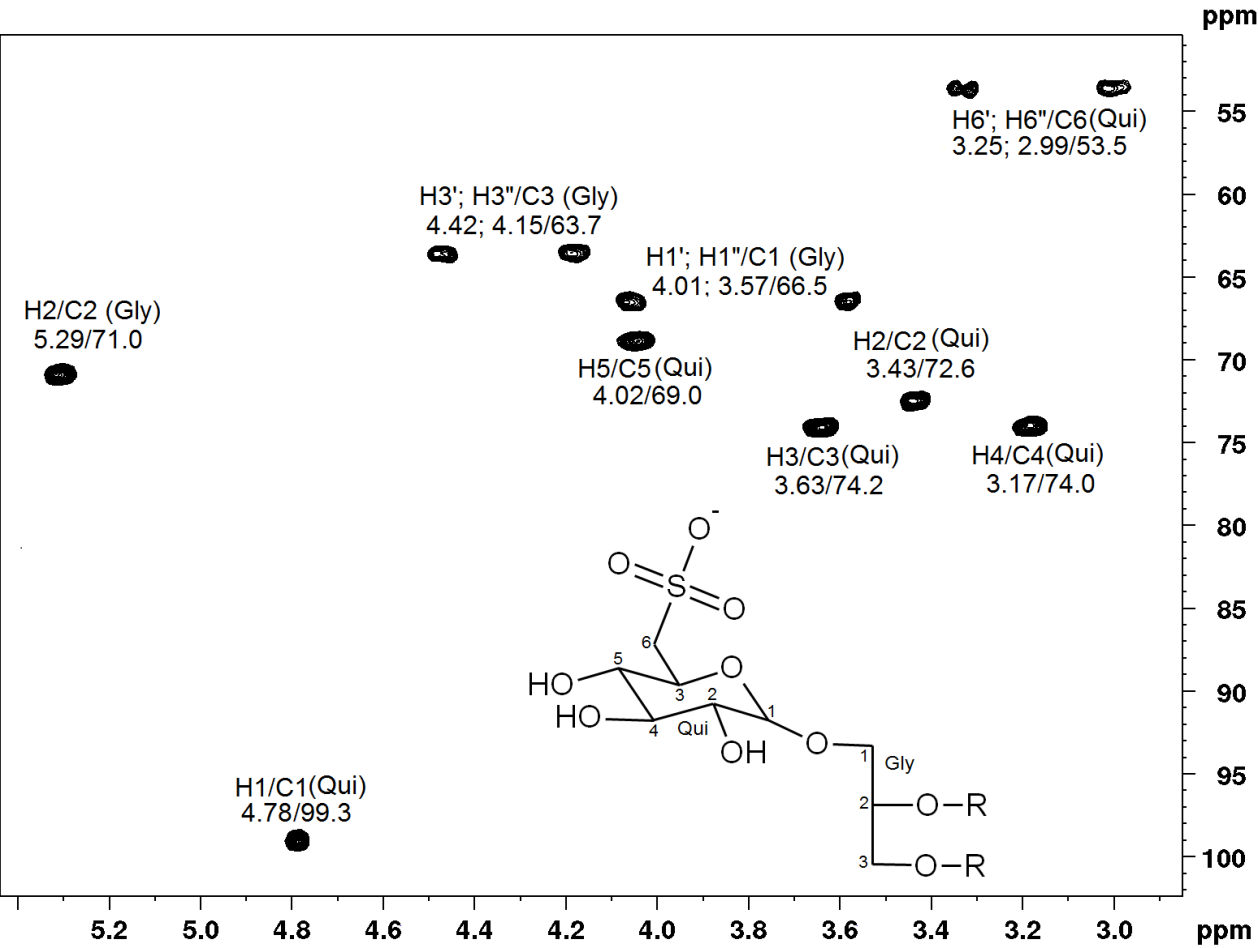
Partial fingerprint spectrum 2D-^1^H/^13^C-HSQC analysis of the polar head group of sulfoquinovosyldiacylglycerol. Gly = glycerol; Qui = quinovose.

The structure of the main sulfoglycolipid present in fraction F4I86 and F4II90 was confirmed by ^1^H and ^13^C NMR analysis, based on HSQC fingerprints. The anomeric region (H1/C1 Qui) contained a single signal at δ 4.78/99.3, consistent with α-quinovopyranosyl group. Moreover, ^1^H/^13^C-HSBC signals at δ 3.25, 2.990/53.5 were observed (Figure 4). The presence of doublets of CH_2_ signals in a high-field region is characteristic of S-substituted C-6, typical of 6-sulfo-α-quinovopyranosyl unit [14,26,27].

These results and those obtained from mass spectrometry allowed us to identify the main SQDG from fractions F4I86 and F4II90 as 1,2-di-*O*-palmitoyl-3-*O*-(6-sulfo**-**α-d-quinovopyranosyl)-glycerol.

### 2.4. Antiviral Activity

Both fractions F4I86 and F4II90 demonstrated strong antiviral activity against HSV-1 and HSV-2 (Table 2). The percentage of inhibition of fraction F4I86 was 99.9 against HSV-1 and HSV-2. The fraction F4II90 inhibited HSV-1 and HSV-2 with a percentage of 96 and 99.9, respectively.

**Table 2:** Anti HSV-1 and HSV-2 of fractions F4I86 and F4II90 isolated from *S. vulgare*.

Compounds	CC_50_ (μg/mL)	MNTC (μg/mL)	Inhibition %	
			HSV-1	HSV-2
*S. vulgare* F4I86	>200	50	99.9	99.9
*S. vulgare* F4II90	>200	50	96.0	99.9
Acyclovir	>200	200	99.0	99.9

CC_50_, 50% Cytotoxic Concentration; MNTC, Maximum Non-Toxic Concentration; HSV-1, Herpes Simplex Virus 1; HSV-2, Herpes Simplex Virus 2; Acyclovir, standard compound.

Our results are compatible with previous data obtained by de Souza and coworkers [14] who isolated SQDGs with anti-HSV activity from the Brazilian red seaweed *Osmundaria obtusiloba.* Wang and coworkers [21] isolated and purified a SQDG from the *n*-butanol fraction of *Caulerpa racemosa* with anti-HSV2 activity. SQDG with anti-HSV1 activity was isolated from the microalga *Spirulina platensis* [20].

Biological activity from SQDGs may be related to the fatty acids that esterify the sulfoquinosyl moiety [28]. The principal ion present in fractions F4I86 and F4II90 corresponds to the structure of a SQDG esterified by two palmitic acids (C16:0). Such observation is consistent with previous studies that already identified palmitic acid as the most abundant fatty acid present in marine sulfonoglycolipids [18,22,28,29,30,31,32,33,34]. Indeed, palmitic acid is present in all the SQDGs identified in fraction F4II90, and is part of the structure of five of the six SQDGs identified in fraction F4I86.

Palmitic acid as a free fatty acid has been characterized as an antitumor compound present in the red seaweed *Amphiroa zonata* [35], as well as being an antibacterial agent [36]. Santoyo and coworkers [37] suggested that palmitic acid present in extracts from the microalgae *Dunaliella salina* and *Haematococcus pluvialis* may also be involved in the anti-HSV-1 activity. Recently, Lee and coworkers [38] demonstrated that palmitic acid purified from *Sargassum fusiforme* extract binds to the CD4 cell receptor, blocking HIV-1 entry and infection.

Another possible explanation for the antiviral activity of SQDGs is based on the presence of the sulfonate group. As reported by Spear [39], the envelope of HSV contains five glycoproteins (gB, gC, gD, gH and gL) that participate in viral entry by binding to specific receptors present on the cell surface. Within these receptors, heparan sulfate can bind to gB or gC, facilitating the binding of viral glycoproteins to other host cell receptors and allowing the fusion of viral envelop with cell membrane. Such interaction between heparan sulfate and viral glycoproteins may be perturbed by the presence of SQDGs. Indeed, the negatively charged sulfonate group of the SQDGs may interact with positively charged protein sites, therefore explaining the antiviral activity exhibited by these sulfolipids. The relation between the degree of sulfonation and antiviral activity has been highlighted in marine polysaccharides [40]. However, the strong HSV antiviral activity that characterizes marine sulfated polysaccharides, particularly marine heparinoid polysaccharides, is also related to the close structural analogy between these compounds and the heparin sulfate cell receptor [41]. In the case of SQDGs no such analogy exists, and future studies will be necessary to investigate how sulfolipids may interfere in the binding between viral glycoprotein and heparin sulfate cell receptors.

## 3. Experimental Section

### 3.1. Biological Material

Thalli of *S. vulgare* were collected by free diving in the shallow subtidal zone from Ilha de Itacuruçá, a large nearshore island inside Sepetiba Bay (Mangaratiba district, Rio de Janeiro State, Southeastern Brazil—22°56′ S, 43°52′ W). After collection, specimens of *S. vulgare* were immediately transferred to the laboratory in isothermic boxes filled with local seawater, where they were gently washed in seawater, sorted, and carefully cleaned from associated biota. Thalli were then freeze-dried and ground to a fine powder before performing extraction.

### 3.2. Extraction and Fractionation of Lipids

The powder obtained from *S. vulgare* freeze-dried specimens was successively extracted at room temperature with chloroform/methanol 2:1 and 1:2 (v/v). After filtration, the extracts were combined, dried and the crude lipid extract was partitioned according to Folch and coworkers [24]. The lipids recovered from the Folch lower phase were fractionated on a silica gel column, which was eluted with chloroform, acetone and methanol, giving rise to fractions F1–F4. Fraction F4, eluted with methanol and enriched in sulfatides was further purified on a silica gel column, which was sequentially eluted with chloroform/methanol with increasing concentrations of methanol (95:5, 90:10, 80:20, 50:50 v/v) and finally 100% methanol. The resulting fractions were combined in twelve final fractions. Fraction F4I86 was guarded for further analyses and fraction F4I90, eluted with 80/20 chloroform/methanol, was further purified on a second silica gel column yielding a purified sulfolipid fraction, F4II90.

All the fractions were analyzed by TLC developed with CHCl_3_:CH_3_OH: 2 M NH_4_OH (40:10:1 v/v) and the spots visualized with iodine and by spraying with orcinol/H_2_SO_4_ [42].

### 3.3. Mass Spectrometry

The samples were prepared in MeOH at 1 mg/mL, then diluted to 0.1 mg/mL in MeOH-H_2_O (7:3, v/v) and direct infused into ESI source, at a flow rate of 10 μL/min, following the protocol described by de Souza and coworkers [14]. The MS analysis was carried out in an electrospray ionization mass spectrometry (ESI-MS), model Quattro-LC (Waters) with a triple-quadrupole mass analyzer, operating at atmospheric pressure ionization (API), assisted by a syringe pump (Model KDS-100-CE, KD Scientific, Holliston, MA, USA) for sample infusion. Nitrogen was used as nebulizing and desolvation gas and the ionization energies were 50 V on the cone and 2 kV on the capillary, operating in the negative ionization mode. The second stage tandem-MS was obtained by collision induced dissociation mass spectrometry (CID-MS) using argon as collision gas and collision energies ranging between 35 and 60 eV.

### 3.4. Nuclear Magnetic Resonance

NMR analyses were performed on a Bruker Avance III 400 MHZ spectrometer with a 5 mm inversed gradient probe. The samples were dissolved in deuterated chloroform and methanol (1:1, v/v) at 20 mg/mL. Two-dimensional homo- and heteronuclear ^1^H/^13^C correlation experiments (HSQC) were developed. The chemical shifts (δ = ppm) were obtained on the basis of tetramethylsilane shifts (δ13C = 0; δ1H = 0) [14].

### 3.5. Cells and Viruses

Vero cells (African green monkey kidney) were grown in Eagle’s minimum essential medium (Eagle-MEM) and supplemented with 10% (v/v) fetal bovine serum, glutamine (2 mM), garamycin (50 μg/mL), fungizone (amphotericin B) (2.5 μg/mL), NaHCO_3_ (0.25%) and HEPES (10 mM). HSV-1 and HSV-2 were isolated from a typical lip and genital lesion respectively, in the Virology Department of the Federal University of Rio de Janeiro (UFRJ), Brazil. Viruses were typed by polymerase chain reaction (PCR) using specific primers for identification [14,43].

### 3.6. Cytotoxicity Assay

The cytotoxicity of glycolipids was performed by incubating triplicate Vero cell (African green monkey kidney cell) line monolayers cultivated in 96-well microplates with two-fold serial dilutions (200–3.1 μg/mL) of the SQDG fractions for 48 h at 37 °C in a 5% CO2 atmosphere. Cellular viability was evaluated by the neutral red dye-uptake method [44]. The 50% cytotoxic concentration (CC50) was defined as the SQDG concentration, which caused a 50% reduction in the number of viable cells.

### 3.7. Antiviral Activity Assay

The antiviral activity of SQDG and acyclovir was evaluated by the titer reduction. The virus titers were calculated using the Reed and Muench statistical method [45] and expressed as 50% tissue culture infective dose (TCID50) per mL. Vero cell monolayers were treated with the SQDG and acyclovir at the MNTC and 100 TCID50/mL of HSV-1 or HSV-2 suspensions were added to treated and untreated cell cultures and incubated at 37 °C for 48 h in a 5% CO_2_ atmosphere. After incubation, the supernatant was collected and virus titers in treated and untreated cells were determined. The antiviral activity was expressed as percentage of inhibition (PI) [46] using antilogarithmic TCID50 values as follows: PI = [1 − (antilogarithmic test value/antilogarithmic control value)] × 100.

## 4. Conclusions

Antiviral SQDGs were isolated and characterized for the first time in *Sargassum vulgare* from Brazil. Other studies already highlighted antifouling, anticoagulant, antithrombotic, antioxidant and anti-inflammatory activities from *S. vulgare* extracts and isolated compounds. Our results reinforce the potential of *S. vulgare* as a source of natural products with biotechnological applications. Future studies will be necessary to understand more precisely the mechanism of action of SQDGs and to fully determine the potential applications of these seaweed compounds.

## References

[1] Vo T., Ngo D., Ta Q.V., Kim S. (2011). Marine organisms as a therapeutic source against herpes simplex virus infection. Eur. J. Pharm. Sci..

[2] Brady R.C., Bernstein D.I. (2004). Treatment of herpes simplex virus infections. Antiviral. Res..

[3] Celum C.L. (2004). The interaction between herpes simplex virus and human immunodeficiency virus. Herpes.

[4] Bacon T.H., Levin M.J., Leary J.J., Sarisky R.T., Sutton D. (2003). Herpes simplex virus resistance to acyclovir and penciclovir after two decades of antiviral therapy. Clin. Microbiol. Rev..

[5] Morfin F., Thouvenot D. (2003). Herpes simplex virus resistance to antiviral drugs. J. Clin. Virol..

[6] Maschek J.A., Baker B.J., Amsler C.D. (2008). The chemistry of algal secondary metabolism. Algal Chemical Ecology.

[7] Chakraborty S., Ghosh U. (2010). Oceans: A store house of drugs—a review. J. Pharm. Res..

[8] Blunt J.W., Copp B.R., Keyzers R.A., Munro M.H.G., Prinsep M.R. (2012). Marine natural products. Nat. Prod. Rep..

[9] Kim S.K., Karadeniz F. (2011). Anti-HIV activity of extracts and compounds from marine algae. Adv. Food. Nutr. Res..

[10] Saha S., Navid M.H., Bandyopadhyay S.S., Schnitzler P., Ray B. (2012). Sulfated polysaccharides from *Laminaria angustata*: Structural features and *in vitro* antiviral activities. Carbohydr. Polym..

[11] Bandyopadhyay S.S., Navid M.H., Ghosh T., Schnitzler P., Ray B. (2011). Structural features and *in vitro* antiviral activities of sulfated polysaccharides from *Sphacelaria indica*. Phytochemistry.

[12] Cardozo F.T., Camelini C.M., Mascarello A., Rossi M.J., Nunes R.J., Barardi C.R., de Mendonça M.M., Simões C.M. (2011). Antiherpetic activity of a sulfated polysaccharide from *Agaricus brasiliensis* mycelia. Antivir. Res..

[13] Adhikari U., Mateu C.G., Chattopadhyay K., Pujol C.A., Damonte E.B., Ray B. (2006). Structure and antiviral activity of sulfated fucans from *Stoechospermum marginatum*. Phytochemistry.

[14] de Souza L.M., Sassaki G.L., Romanos M.T., Barreto-Bergter E. (2012). Structural characterization and anti-HSV-1 and HSV-2 activity of glycolipids from the marine algae *Osmundaria obtusiloba* isolated from Southeastern Brazilian coast. Mar. Drugs.

[15] Kind T., Meissen J.K., Yang D.W., Nocito F., Vaniya A., Cheng Y.S., VanderGheynst J.S., Fiehn O. (2012). Qualitative analysis of algal secretions with multiple mass spectrometric platforms. J. Chromagraph. A.

[16] Packter N.M. (1985). Lipids in plants and microbes: By J L Harwood and N J Russel. pp 162. George Allen & Unwin, London. 1984. Biochem. Educ..

[17] Morimoto T., Murakami N., Nagatsu A., Sakakibara J. (1993). Studies on glycolipids VII. Isolation of two new sulfoquinovosyl diacylglycerols from green alga *Chlorella vulgaris*. Chem. Pharm. Bull..

[18] Gustafson K.R., Cardellina J.H., Fuller R.W., Weislow O.S., Kiser R.F., Snader K.M., Patterson G.M.L., Boyd M.R. (1989). AIDS-antiviral sulfolipids from cyanobacteria (Blue-Green Algae). J. Natl. Cancer Inst..

[19] Kikuchi H., Tsukitani Y., Manda T., Fujii T., Nakanishi H., Kobayashi M., Kitagawa I. (1982). Marine Natural Products. X. Pharmacologically active glycolipids from the Okinawan marine sponge *Phyllospongia foliascens* (PALLAS). Chem. Pharm. Bull..

[20] Chirasuwan N., Chaiklahan R., Kittakoop P., Chanasattru W., Ruengjitchatchawalya M., Tanticharoen M., Bunnag B. (2009). Anti HSV-1 activity of sulphoquinovosyl diacylglycerol isolated from *Spirulina platensis*. Scienceasia.

[21] Wang H., Li Y.L., Shen W.Z., Rui W., Ma X.J., Cen Y.Z. (2007). Antiviral activity of a sulfoquinovosyldiacylglycerol (SQDG) compound isolated from the green alga *Caulerpla racemosa*. Bot. Mar..

[22] Khotimchenko S.V. (2002). Distribution of glyceroglycolipids in marine algae and grasses. Chem. Nat. Compd..

[23] Barreto-Bergter E., Sassaki G.L., Souza L.M. (2011). Structural analysis of fungal cerebrosides. Front. Microbiol..

[24] Folch J., Lees M., Sloane-Stanley G.H. (1957). A simple method for the isolation and purification of total lipids from animal tissues. J. Biol. Chem..

[25] Zianni R., Bianco G., Lelario F., Losito I., Palmisano F., Cataldi T.R.I. (2013). Fatty acid neutral losses observed in tandem mass spectrometry with collision-induced dissociation allows regiochemical assignment of sulfoquinovosyl-diacylglycerols. J. Mass Spectrom..

[26] Sassaki G.L., Gorin P.A.J., Tischer C.A., Iacomini M. (2001). Sulfonoglycolipids from the lichenized basidiomycete *Dictyonema glabratum*: Isolation, NMR, and ESI-MS approaches. Glycobiology.

[27] Souza L.M., Iacomini M., Gorin P.A.J., Sari R.S., Haddad M.A., Sassaki G.L. (2007). Glyco- and sphingophosphonolipids from the medusa *Phyllorhiza punctata*: NMR and ESI-MS/MS fingerprints. Chem. Phys. Lipids.

[28] Tsai C.J., Pan B.S. (2012). Identification of sulfoglycolipid bioactivities and characteristic fatty acids of marine macroalgae. J. Agric. Food Chem..

[29] Al-Fadhli A., Wahidulla S., D’Souza L. (2006). Glycolipids from the red alga *Chondria armata* (Kutz.) Okamura. Glycobiology.

[30] Fusetani N., Hashimoto Y. (1975). Structures of two water-soluble hemolysins isolated from green alga *Ulva pertus*. Agric. Biol. Chem..

[31] Araki S., Sakurai T., Oohusa T., Kayama M., Sato N. (1989). Characterization of sulphonoquinovosyl diacylglycerol from marine red alga. Plant Cell Physiol..

[32] Son W.B. (1990). Glycolipids from *Gracilaria verrucosa*. Phytochemistry.

[33] Siddantha A.K., Ramvat B.K., Chauvan V.D., Achari B., Dutta P.K., Pakrashi S.C. (1991). Sulphoglycolipid from the green alga *Enteromorpha flexuosa* (Wulf). J. Agric. Bot. Mar..

[34] Logvinov S.V., Denisenko V.A., Dmitrenok P.S., Moiseenko O.P. (2012). Sulfoquinovosyldiacylglycerins from *Scaphechinus mirabilis*. Chem. Nat. Compd..

[35] Harada H., Yamashita U., Kurihara H., Fukushi E., Kawabata J., Kamei Y. (2002). Antitumor activity of palmitic acid found as a selective cytotoxic substance in a marine red alga. Anticancer Res..

[36] Kabara J.J., Swieczkowski D.M., Truant J.P., Conley A.J., Truant J.P. (1972). Fatty-acids and derivatives as antimicrobial agents. Antimicrob. Agents Chemother..

[37] Santoyo S., Jaime L., Plaza M., Herrero M., Rodriguez-Meizoso I., Ibañez E., Reglero G. (2012). Antiviral compounds obtained from microalgae commonly used as carotenoid sources. J. Appl. Phycol..

[38] Lee D.Y., Lin X., Paskaleva E.E., Liu Y., Puttamadappa S.S., Thornber C., Drake J.R., Habulin M., Shekhtman A., Canki M. (2009). Palmitic acid is a novel CD4 fusion inhibitor that blocks HIV entry and infection. AIDS Res. Hum. Retrovir..

[39] Spear P.G. (2004). Herpes simplex virus: Receptors and ligands for cell entry. Cell Microbiol..

[40] Wang W., Wang S.X., Guan H.S. (2012). The antiviral activities and mechanisms of marine polysaccharides: An Overview. Mar. Drugs.

[41] Neyts J., Snoeck R., Schols D., Balzarini J., Esko J.D., Van Schepdael A., De Clercq E. (1992). Sulfated polymers inhibit the interaction of human cytomegalovirus with cell surface heparan sulfate. Virology.

[42] Skipski V.P. (1975). Thin layer chromatography of neutral glycolipids. Methods Enzymol..

[43] Markoulatos P., Georgopoulou A., Siafakas N., Plakokefalos E., Tzanakaki G., Kourea-Kremastinou J. (2001). Laboratory diagnosis of common herpesvirus infections of the central nervous system by a multiplex PCR assay. J. Clin. Microbiol..

[44] Borenfreund E., Puerner J.A. (1985). Toxicity determined *in vitro* by morphological alterations and neutral red absorption. Toxicol. Lett..

[45] Reed L.J., Muench H. (1938). A simple method of estimating 50 per cent end-points. Am. J. Hyg..

[46] Nishimura T., Toku K., Fukuyasu H. (1977). Antiviral compounds. XII. Antiviral activity aminohydrazones of alkoxyphenyl substituted carbonyl compounds against influenza virus in eggs and mice. Kitasato Arch. Exp. Med..

